# Effects of Moderate-Volume, High-Load Lower-Body Resistance Training on Strength and Function in Persons with Parkinson's Disease: A Pilot Study

**DOI:** 10.4061/2010/824734

**Published:** 2010-03-16

**Authors:** Brian K. Schilling, Ronald F. Pfeiffer, Mark S. LeDoux, Robyn E. Karlage, Richard J. Bloomer, Michael J. Falvo

**Affiliations:** ^1^Exercise Neuromechanics Laboratory, The University of Memphis, Memphis, TN 38152, USA; ^2^Department of Neurology, The University of Tennessee Health Science Center, Memphis, TN 38163, USA; ^3^Movement Science Program, Washington University in St. Louis, MO 63108, USA

## Abstract

*Background*. Resistance training research has demonstrated positive effects for persons with Parkinson's disease (PD), but the number of acute training variables that can be manipulated makes it difficult to determine the optimal resistance training program. *Objective*. The purpose of this investigation was to examine the effects of an 8-week resistance training intervention on strength and function in persons with PD. *Methods*. Eighteen men and women were randomized to training or standard care for the 8-week intervention. The training group performed 3 sets of 5–8 repetitions of the leg press, leg curl, and calf press twice weekly. Tests included leg press strength relative to body mass, timed up-and-go, six-minute walk, and Activities-specific Balance Confidence questionnaire. *Results*. There was a significant group-by-time effect for maximum leg press strength relative to body mass, with the training group significantly increasing their maximum relative strength (*P* < .05). No other significant interactions were noted (*P* > .05). *Conclusions*. Moderate volume, high-load weight training is effective for increasing lower-body strength in persons with PD.

## 1. Introduction

Individuals with Parkinson's disease (PD) exhibit deficiencies in the ability of the neuromuscular system to produce force [1], including both its rate [2–5] and magnitude [4–9]. Mechanisms are not clearly understood, albeit some investigators have suggested an inability to fully activate the motor neuron pool [10]. As a result, strength deficits appear to be at least partially central in nature, as PD drug therapy [2, 11] and deep brain stimulation [12] improve neuromuscular performance. Loss of muscle mass may also be a mechanism by which weakness is caused [13], even though strength relative to body mass has been shown to be lower in PD [14].Irrespective of mechanism, impaired force production, or muscle weakness, is a common symptom of PD [15], a secondary cause of bradykinesia [16], and a contributing factor in postural instability [17]. These effects, along with the obvious associations with functional mobility [14] and quality of life, dramatically underscore the need to develop interventions that ameliorate these impairments.

Recent reviews of literature stressed the efficacy of exercise interventions in treating the symptoms for persons with PD, but questions were posed as to the optimal exercise prescription [18, 19]. Falvo et al. [1] highlighted the potential benefits of resistance training in particular, but little research has been done to determine the optimal resistance training program for persons with PD, particularly in terms of exercise volume. The resistance training programs utilized in these few studies to date have varied widely due to the number of acute training variables such as repetitions per set, number of sets, mode of weight training, rest periods, and frequency. However, even with the variation in exercise programming and testing procedures, resistance training interventions have been effective in treating the symptoms of PD [1]; increases in strength [20–22], gait velocity [23], and functional mobility have been noted [20, 21, 24].

The purpose of this investigation was to examine the effects of a moderate volume, high-load 8-week resistance training intervention on lower-body strength and functional mobility in persons with mild to moderate PD, and to examine the relationship of strength to neuromuscular function. Our research is novel in that we structured the program around a multijoint extensor action (leg press) as extensor actions are disproportionately affected by PD [25], and multijoint exercises more closely mimic the activities of daily living (ADL). Repetition range was considerably lower (implying greater load) than extant studies. The exercises were isoinertial (i.e., constant gravitational load), also similar to the resistance encountered in ADL. We also used a 3-set per exercise training volume that is proposed to be superior to one-set programs in older populations [26, 27], but has not been used in persons with PD.

## 2. Methods

### 2.1. Participants

Eighteen participants with mild to moderate PD were randomized to either a training (6 men, 3 women) or standard care control group (5 men, 4 women). All participants provided written informed consent as approved by the University Institutional Review Board. All participants were carefully screened to have the ability to walk a 20-foot path, turn, and return to the start without use of an assistive device. Subjects were not participating in a structured exercise program, and were instructed to maintain their normal activities for the duration of the study. Participants with orthostatic hypotension, dementia (Mini-Mental State Examination Scores < 24), or other significant comorbidities (i.e., stroke, musculoskeletal problems in the lower extremity) were not recruited into the study. Inclusion criteria included primary PD with a Hoehn and Yahr stage of 1–2.5 when in an “on” state of medication. All participants reported to the lab in their optimally medicated state for both testing and training (typically within 30 min to 2 hours of their first morning dose), and none were receiving deep-brain stimulation.

### 2.2. Testing Procedures

**Figure 1:**
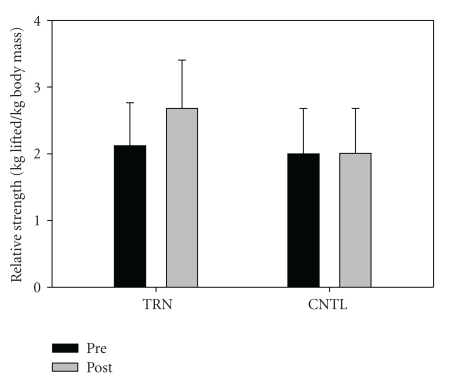
Leg Press 1-RM adjusted for body mass (kg/kg) for TRN (Pre = 4.7 ± 1.4, Post 5.9 ± 1.6) and CNTL (Pre = 4.4 ± 1.5, Post 4.4 ± 1.4). A significant interaction effect was noted (*P* = .001). Post hoc analysis indicates a significant increase over time for TRN (*P* = .001).

Testing included a battery of strength and functional measures. Maximum strength for the lower-body was assessed using a leg press machine (Hammer Strength, Figure 1). The maximum weight lifted, after warm-up, for one repetition was recorded as the one repetition maximum (1-RM), in accordance with the methods of Verdijk et al. [28]. This weight was then divided by body mass to generate a measure of relative strength. We propose this measure to be a good indicator of tasks that involve moving one's own body mass such as rising from a chair [6]. Strength data from the other training exercises were not recorded. 

The Activities-specific Balance Confidence (ABC) scale was used to evaluate participants' confidence level of daily activities [29]. The percentage scores of 16 questions were summed to yield a single ABC score for data analysis. The higher the score, the more confident a participant perceives their balance abilities. This scale has been shown to be a good indicator of balance ability and a predictor of fall likelihood [30, 31]. The ABC scale has been validated in PD [32].

Timed up-and-go was administered as an indicator of ADL ability [33]. Participants were instructed to rise from a chair (without using their arms) on a verbal cue, walk three meters to a cone at a comfortable pace without running, and return to the chair. The best time from the rise to touch down for two trials was used for analysis and a harness system (Solostep) was available if needed to ensure safety of participants during testing. Functional capacity was assessed using the 6-minute walk test (6MWT). The 6MWT is a standard test used in clinical settings for measuring the efficacy of therapeutic interventions [34]. The total distance (m) was measured as participants walk for a period of six minutes. 

After baseline testing, participants were gender-match randomly assigned to either training or control groups for the eight-week duration of the study. Controls were instructed to continue their standard care, and were given an opportunity to complete the training intervention after they completed initial eight-week control period. The training group performed, after warm-up, three sets of 5–8 repetitions for the leg press, seated leg curl, and calf press (Hammer Strength) twice weekly under direct supervision from a Certified Strength and Conditioning Specialist. Initial training weight was established via trial and error in the first and second session, so that each subject was performing at least two sets for 8 repetitions, and the final set between 5–8 repetitions. Each set was a maximal effort carried out to volitional fatigue. Subjects were instructed to lift the weight as fast as possible with good form and to slowly return the weight to the start position. The leg press device was configured by the manufacturer so that each leg moved independently, thus preventing possible masking of the weaker leg by the more dominant leg if asymmetry existed. Progression was planned so that when eight repetitions could be completed for all three sets, the weight was increased 5%–10%, so that each subject was performing at least two sets for 8 repetitions, and the final set between 5–8 repetitions.

### 2.3. Data Analyses

Effects of the intervention were analyzed using a 2 × 2 (group by time) repeated measures analysis of variance. When interaction was noted, Tukey post hoc tests were used to determine group changes from baseline. Statistical significance was set a priori at *P* ≤ .05. Effect size measures (ES) were used to show changes in terms of standard deviation.

## 3. Results

**Figure 2:**
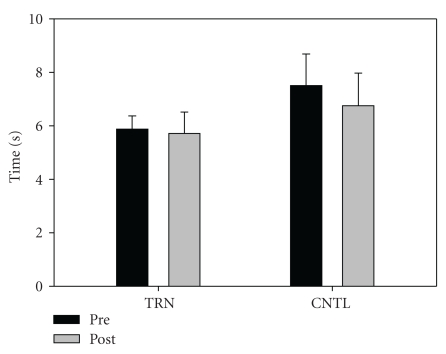
TUG (s) for TRN (Pre = 5.8 ± 0.50, Post 5.7 ± 0.80) and CNTL (Pre = 7.5 ± 1.18, Post 6.75 ± 1.21). No significant interaction (*P* = .223) or time effect (*P* = .069) was noted.

**Figure 3:**
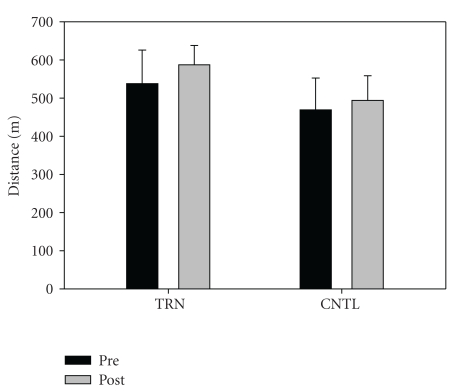
Six minute walk distance (m) for TRN (Pre = 537.7 ± 88.1, Post 586.9 ± 51.0) and CNTL (Pre = 468.8 ± 83.3, Post 493.9 ± 64.3). No significant interaction was noted (*P* = .296), but a significant time effect was noted (*P* = .005).

**Figure 4:**
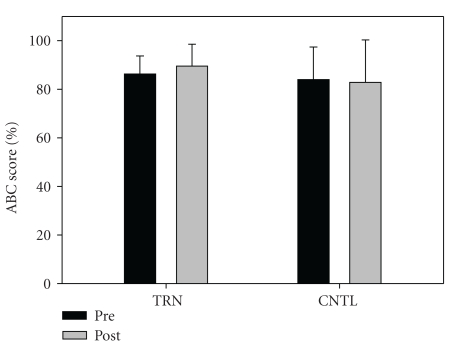
ABC scores (%) for TRN (Pre = 86.2 ± 7.5, Post 89.5 ± 9.0) and CNTL (Pre = 83.9 ± 13.4, Post 82.8 ± 17.5). No significant interaction was noted (*P* = .381).

Fifteen subjects completed the intervention. One individual from the training group and two in the control group opted not to complete the study for personal/physical reasons unrelated to the study itself. Subject descriptive data are shown in Table 1. No significant differences between groups were noted at baseline. There was a significant group-by-time interaction for relative (*P* = .001) and absolute (*P* = .001) leg press strength. Training resulted in a significant increase in relative leg press (kg/kg) 1-RM (Pre = 2.1 ± 0.6, Post = 2.7 ± 0.7), *P* < .001, ES = 0.9, Figure 1). Absolute strength also increased significantly in the training group (Pre = 161.0 ± 66.9 kg, Post = 199.9 ± 67.8 kg, *P* < .001, ES = 0.6). In contrast, no changes were noted in relative strength for the control group (Pre = 2.0 ± 0.7, Post = 2.0 ± 0.7. Data from the control group indicate that strength appears stable over the short term (8 weeks) in persons with PD. No significant interaction (*P* = .223) or time effect (*P* = .07) was noted for TUG (TRN: Pre = 5.8 ±  0.50, Post = 5.7 ± 0.80, ES = 0.2, CNTL: Pre = 7.5 ± 1.2, Post = 6.8 ± 1.2, ES = 0.6, Figure 2). No significant interaction was noted (*P* = .296), but a significant time effect was noted (*P* = .005) for six minute walk distance (Pre = 503.3 ± 85.7, Post 540.4 ± 57.7, ES = 0.5 Figure 3). No significant interaction (*P* = .381) or time effect (*P* = .664) was noted for ABC scores (TRN; Pre = 86.2 ± 7.5, Post 89.5 ± 9.0, ES = 0.4, CNTL; Pre = 83.9 ± 13.4, Post 82.8 ± 17.5, ES = 0.1, Figure 4).

## 4. Discussion

This is the first investigation to date that utilized a moderate volume (three sets of three exercises) resistance training program consisting of multijoint exercises performed for the lower body muscles. The only other study to use multiple sets used a load of only 60% of a 4-repetition maximum, and used only single-joint exercises [35]. Our results indicate that this volume of resistance training had positive effects on muscle strength in this population, and was well tolerated. No improvements were noted for the functional measures in this investigation, but it should be understood that our sample of subjects performed well at baseline for all the functional measures and thus may have had limited potential for gains in those measures. Several studies have shown increases in function with strength training in neurologically normal older adults [36, 37], and in persons with PD [20, 24]. It may be found that greater gains in strength are needed for improvement in functional measures.

### 4.1. Leg Strength

The percent increase in our strength measures (relative 29%, absolute, 24%, *P* = .001) is somewhat greater than the percent increase noted in most of other existing investigations. Studies have shown 18% improvement in unilateral knee extension torque [35], as well as 18% [20] and 28% [24] gain in unilateral isometric knee extension strength of the more affected limb. Hass et al. [21] demonstrated increases in knee extension strength of 16% and 18% in the control and creatine-supplemented groups of their study. However, Hirsch et al. [22] showed 44% increase in four-repetition knee extension strength, but this task was not a maximal test. The four-repetition test is unique to that study and is somewhat absent as a performance test in strength training literature. Our maximum strength measure, presented in relation to the load most often encountered in ADL (body mass), may be a better indicator of overall functional ability [38] than non-adjusted strength, and has been shown to correlate with TUG [14]. It may be important in future investigations to adjust lower-body strength measures to lower-body muscle mass in order to have a more precise estimation of muscle function.

Most researches related to resistance training in PD have utilized very low-volume interventions [21–23]. Moreover, the majority of these studies have employed exercises that target a single-joint [21, 22, 35]. Some have used novel interventions such as eccentric loading [20, 24] or the addition of nutritional supplements known to augment strength gains in neurologically normal adults [21]. Additionally, there has been wide disparity in the mode of strength assessment, and in many times the testing mode is not specific to the training mode. Such discrepancies may mask the training effect [39]. Strength testing modes include isokinetic (constant velocity) [35], isoinertial (constant load) [23], and isometric performance [20, 24], most often of a single joint. 

Although comparing the effects of a moderate exercise volume without a direct comparison to lower-volume of weight training is problematic, our results do indicate that a three-set volume is as least are effective in increasing strength, and possibly more based on extant studies. In addition, our strength measure is limited by the weaker leg, as the arms of the leg press device move independently, and only repetitions where both legs were extended were used for analysis. As such, comparison to the data of Dibble et al. [20, 24] where limbs were measured independently seems reasonable. However, their strength measure was an isometric contraction and single joint in nature, compared to the bilateral, dynamic, multijoint action utilized in our study.

### 4.2. Functional Mobility

The fact that leg strength increases did not parallel changes in functional mobility in this investigation was unexpected, but may suggest that the strength training adaptations may not carry over to mobility tasks in the short term. It is likely that the baseline characteristics of our study population also contributed to this finding. In particular, our subjects, mean TUG (6.7 s) was considerably faster than normative data for 60–69-year-old individuals (8.1 s) [40]. Other investigations have shown significant performance increases in such measures as 10-minute walk velocity and TUG [20], as well as six-minute walk and stair descent time [24], but the participants in these investigations were slightly more affected based on Hoehn and Yahr scores (2.5 compared to ~2.0 in our study). Considering the findings of these studies, and since correlations have been found between strength and TUG [14], and with TUG and fall risk [41], further research on the effect of strength training on functional mobility tests such as timed walking is warranted.

The significant time effect for the 6MWT suggests a learning effect [42] that was unexpected by the research team. It is possible that a long testing period caused the subjects to underestimate their ability for the test during the first trial, leading to an increase during the second trial. Perhaps a practice walk should be performed so that the subjects can gauge their abilities and would be able to put forth the appropriate effort for the testing sessions. Timed walk distance has been shown to improve after strength training in previous studies [20, 43], and its association with other measures of balance (ABC Scores [41], Berg Balance Scale [44]) warrants further investigation. Our subjects again scored rather well on this measure (baseline mean 502 m) compared to extant data of persons with PD (392 m) [44].

### 4.3. ABC

Since the subjects scored well in the ABC questionnaire preintervention, it is not surprising that no interaction was noted. Ours is the first weight training intervention to use such a questionnaire, and it is the author's opinion that such subjective measures of intervention effectiveness should be applied to future research. Another work has shown that ABC scores can predict 17.1% of the variance in the 6MWT distance [45], suggesting that this is an important measure for persons with PD. Addition of the PDQ-39 [46], Fatigue Severity scale [47] and possibly other scales may be useful in evaluating the subjective effectiveness and efficacy of resistance training interventions in the future.

Future studies of resistance training should include subjects with more advanced disease. Additionally, to determine the interaction of resistance training with the progression of PD, interventions should be carried out over a longer duration. Investigation on the mechanism of increased force production including muscle hypertrophy and nervous system adaptation is warranted. Furthermore, work is needed to optimize training variables such as volume, load, mode, rest periods, and repetition number. The ultimate effectiveness of these interventions should be determined via the effects on PD symptoms and those activities of daily living most affected by PD. This study demonstrated significant increases in multijoint isoinertial strength with a moderate volume weight training program, and this volume of exercise was well tolerated in persons with PD.

## Figures and Tables

**Table 1:** Descriptive subject data (mean ±SD).

Variable	Training Men = 5, Women = 3	Control Men = 4, Women = 3
UPDRS Total	19.1 ± 7.0	23.3 ± 18.0
Hoehn and Yahr (on)	Stage 2, *n * = * 7*; Stage 2.5, *n * = * 1 *	Stage 1.5, *n * = * 2*; Stage 2, *n * = * 4*; Stage 2.5, *n * = * 1 *
Age (y)	61.3 ± 8.6	57.0 ± 7.1
Weight (kg)	76.0 ± 24.5	79.2 ± 27.6
